# Oxaliplatin plus raltitrexed and leucovorin-modulated 5-fluorouracil i.v. bolus: a salvage regimen for colorectal cancer patients

**DOI:** 10.1038/sj.bjc.6600414

**Published:** 2002-06-17

**Authors:** P Comella, R Casaretti, E Crucitta, F De Vita, S Palmeri, A Avallone, M Orditura, L De Lucia, S Del Prete, G Catalano, V Lorusso, G Comella

**Affiliations:** Medical Oncology A, National Tumour Institute, Via M. Semmola, 80131 Naples, Italy; Medical Oncology, Oncology Institute, Via G. Amendola 209, 70126 Bari, Italy; Medical Oncology, Second University School of Medicine, Via S. Pansini, 80131 Naples, Italy; Medical Oncology, University School of Medicine, P.za delle Cliniche 2, 90127 Palermo, Italy; Medical Oncology, City Hospital, Via Tescione 81, 81100 Caserta, Italy; Medical Oncology, City Hospital, Via Pirozzi, 80027 Frattamaggiore, Italy

**Keywords:** oxaliplatin, ralitrexed, 5-fluorouracil, colon carcinoma, salvage regimen

## Abstract

The aim of the present study was to define the activity and tolerability of a triplet regimen including oxaliplatin 130 mg m^−2^ (2 h i.v. infusion) and raltitrexed 3.0 mg m^−2^ (15 min i.v. infusion) given on day 1, followed by levo-folinic acid 250 mg m^−2^ (2 h i.v. infusion) and 5-fluorouracil 1050 mg m^−2^ i.v. bolus on day 2, every 2 weeks, in pretreated colorectal cancer patients. From April 1999 to December 2000, 50 patients were enrolled: 26 were males and 24 females, their median age was 63 (range, 43–79) years; ECOG performance status was 0 in 26 patients, ⩾1 in 24 patients; 26 patients had received previous adjuvant chemotherapy, 40 patients had been exposed to one or two lines of palliative chemotherapy (including irinotecan in 31 cases); 18 patients were considered chemo-refractory. A total of 288 cycles were administered, with a median number of 6 (range 1–12) courses per patient. A complete response was obtained in three patients, and a partial response in nine patients, giving a major response rate of 24% (95% confidence interval, 13–38%), while 15 further patients showed a stable disease, for an overall control of tumour growth in 60% of patients. Three complete responses and three partial responses were obtained in patients pretreated with irinotecan (response rate, 19%); among refractory patients, three achieved partial responses (response rate, 13%). After a median follow-up of 18 (range, 10–30) months, 40 patients showed a progression of disease: the growth modulation index ranged between 0.2 and 2.5: it was ⩾1.33 (showing a significant delay of tumour growth) in 16 (40%) patients. Actuarial median progression-free survival time was 7.6 months, and median survival time was 13.6 months: estimated probability of survival was 55% at 1 year. Main severe toxicity was neutropenia: World Health Organisation grade 4 affected 32% of patients; non-haematological toxicity was mild: World Health Organisation grade 3 diarrhoea was complained of by 8%, and grade 3 stomatitis by 4% of patients; neurotoxicity (according to Lévi scale) was scored as grade 3 in 8% of patients. In conclusion, this regimen was manageable and active as salvage treatment of advanced colorectal cancer patients; it showed incomplete cross-resistance with irinotecan-based treatments, and proved to delay the progression of disease in a relevant proportion of treated patients.

*British Journal of Cancer* (2002) **86**, 1871–1875. doi:10.1038/sj.bjc.6600414 www.bjcancer.com

© 2002 Cancer Research UK

## 

Until the recent introduction into clinical practice of novel active agents against colorectal carcinoma, 5-fluorouracil (FU) has been the mainstay of treatment for this disease. However, only a few patients show a major response to this drug, with a very modest effect on long-term survival.

For patients not responding to, or progressing after, FU-based front-line therapy, both irinotecan (CPT-11) and oxaliplatin (L-OHP) have been claimed as active in second-line. While CPT-11 has been used as a single agent in this setting, demonstrating to positively affect the symptom control and the probability of survival of FU-refractory patients (Cunningham *et al*, 1998; Rougier *et al*, 1998), scant experience exists about the activity of L-OHP alone in second-line (Machover *et al*, 1996). Indeed, this drug has usually been used in combination with FU (Bleiberg and de Gramont, 1998; Raymond *et al*, 1998a), on the grounds of preclinical observations suggesting that L-OHP has a synergistic antitumour activity with FU in either FU-sensitive or FU-resistant murine leukaemia cell cultures or human colonic xenografts transplanted in nude mice (Fischel *et al*, 1998; Raymond *et al*, 1998b; Taron *et al*, 1999; Plasencia *et al*, 2001). Recently, *in vitro* studies on FU-sensitive and FU-resistant HT29 and LoVo cancer cell lines have demonstrated that the combination of L-OHP+FU induced a significant decrease in thymidylate synthase (TS) expression as compared to the administration of FU as single agent (Plasencia *et al*, 2001).

On the other hand, L-OHP has also been combined with raltitrexed (Tomudex® (TOM)), a direct and specific thymidylate synthase inhibitor. Phase I trials with this combination have already been conducted in Europe, demonstrating that full doses of both agents (i.e., 130 mg m^−2^ of L-OHP and 3.0 mg m^−2^ of TOM) may be safely combined every 3 weeks. This regimen has already been assessed in chemonaive as well in previously treated colorectal cancer patients (Fizazi *et al*, 2000; Scheithauer *et al*, 2001a,b).

Interestingly, TOM+FU association has also been shown to exert additive or synergistic cytotoxic effects *in vitro*. These findings may be explained by the observation that, although the two drugs share the TS enzyme as the common target, they inhibit it through different binding sites: polyglutamated TOM binds the folate-binding site, while FdUMP (the active metabolite of FU) binds the pyrimidine-binding (i.e., the catalytic) site. Interestingly, *in vitro* studies have shown a synergistic activity in HCT-8 colon carcinoma cell line with a 24 h TOM exposure followed by a short (4 h) FU exposure, while a marginal synergy was obtained with the same sequence but with a prolonged (5-day) FU exposure (Longo *et al*, 1998). In addition, a phase I study has demonstrated a positive pharmacokinetic interaction between the two drugs, because a TOM dosage ⩾2.5 mg m^−2^ significantly increased the C_max_ and the AUC of the following administration of FU (Dragnev *et al*, 1998).

To elucidate the role of the addition of folinic acid (FA) to this combination, we have carried out a series of experiments on colon and head and neck cancer cell lines, exposed to different concentrations of TOM alone, or FU plus *levo*-FA (LFA), or TOM combined with FU+LFA, given either simultaneously or with a 24 h interval. A synergism between TOM and FU+LFA was observed when TOM preceded FU+LFA in all but one cell line, while the concomitant exposition reduced the inhibition of cell growth as compared to TOM alone. The potentiation factor of the addition of LFA to TOM+FU combination was greater than 1 (ranging from 1.2 to 2) in all cell lines, clearly showing a positive effect of this addition (Caponigro *et al*, 2001). These *in vitro* studies were paralleled by a clinical trial obtaining a 24% response rate in advanced colorectal cancer patients with a sequential administration of TOM and leucovorin-modulated FU given 24 h apart (Comella *et al*, 2000b).

In consideration of the growing proportion of colorectal cancer patients that are exposed to combination regimens, including modulated-FU and either CPT-11 or L-OHP, in the adjuvant setting as well as in the front-line treatment for the advanced disease, there is an urgent need to develop alternative regimens for early relapsing or progressing patients. For this reason, we have decided to explore the biweekly administration of L-OHP and TOM plus FU+LFA in pretreated colorectal cancer patients. We have carried-out a phase I study with this new triplet, concluding that L-OHP 130 mg m^−2^, and TOM 3.0 mg m^−2^ may be given together on day 1, followed 24 h later by a short infusion of LFA 250 mg m^−2^ plus FU 1050 mg m^−2^ given as i.v. bolus; at these recommended doses, severe neutropenia was the main acute toxicity, affecting 31% of patients, while other extra-haematological adverse events were quite infrequent (Comella *et al*, 2000a). The encouraging response rate observed in heavily pretreated patients prompted us to further assess the activity and tolerability of this regimen in the present phase II trial.

## MATERIALS AND METHODS

### Patient selection

Patients affected by histologically proven colorectal carcinoma were eligible for this study. At least one measurable indicator lesion was required. All patients must have been pretreated with FU-based regimen, either as adjuvant or palliative chemotherapy, and previous treatment should have been discontinued for at least 4 weeks. In addition, a good bone marrow reserve was required, with an absolute neutrophil count (ANC) ⩾1500 per mm^3^, a platelet (PLT) count ⩾100 000 per mm^3^, and haemoglobin level ⩾9.5 g dl^−1^; in absence of liver metastases, bilirubin serum level should have been ⩽1.5 × upper normal limit (UNL), and ASAT and ALAT ⩽2.5 × UNL. Exclusion criteria were: presence of brain metastases; poor performance status (⩾3 of the ECOG scale); a life expectancy <12 weeks; uncontrolled metabolic disorders or active infections. Written informed consent was required from each patient before the admission to this trial, which was approved by the Independent Ethical Committee of the National Tumour Institute of Naples.

### Treatment

Patients were submitted to a biweekly regimen consisting of L-OHP 130 mg m^−2^ diluted in 500 ml of 5% DW solution given i.v. over 2 h, followed by TOM 3.0 mg m^−2^ as short (15 min) i.v. infusion on day 1; on the day 2, LFA 250 mg m^−2^ was administered as 2 h i.v. infusion, at the end of which FU 1050 mg m^−2^ was given as i.v. bolus. Cycles were repeated every 2 weeks, in the presence of ANC count ⩾1500 per mm^3^, PLT count ⩾100 000 per mm^3^, and provided that any non-haematological toxicity had recovered to a grade ⩽1. Otherwise, a 1- or 2-week delay was allowed. If toxicity persisted at that time, patients went off study. In the presence of grade 4 haematological toxicity, or in the presence of grade ⩾3 non-haematological toxicity, the subsequent cycles were administered, after recovery, with a 25% dose reduction of all cytotoxic drugs. L-OHP dosage was reduced by 25% only in presence of grade 3 toxicity (persistence of peripheral toxicity at the time of recycling).

### Evaluation of toxicity

For the assessment of acute toxicity, blood cell counts were performed weekly, and twice a week in the case of grade 4 toxicity. Biochemistry was performed before each cycle. History and neurological examination, to detect any sign of neurotoxicity, was performed at initial treatment and at every cycle thereafter. The acute toxicity was graded according to World Health Organisation (WHO) toxicity criteria (Miller *et al*, 1981). Neurotoxicity was scored according to the Lévi scale (Lévi *et al*, 1992).

### Evaluation of response

At study entry, all patients were submitted to routine chemistry, blood cell count, CEA and CA 19.9 serum level determinations, chest X-ray and abdominal ultrasound scan. Indicator lesions were measured with CT or MNR imaging. Endoluminal recurrent or unresected disease was evaluated with fiberoptic endoscopy. WHO criteria were adopted for the evaluation of response (Miller *et al*, 1981). To classify the type of response, all tests which were abnormal at baseline were repeated after every four cycles of treatment.

### Statistical consideration and sample size

We used the statistical design of Gehan (1961) for this phase II study: assuming for the experimental regimen a 15% activity rate, a minimum of 14 patients had to be treated. In case of no major response, the accrual could be stopped, and this hypothesis rejected with a 95% confidence. Otherwise, a number of additional patients, according to the number of responses observed among the first 14, had to be enrolled to estimate the true activity of this regimen.

Time to tumour progression before study entry (TTP_1_) was recorded for each patient. Time to further tumour progression (TTP_2_) was evaluated from the date of study entry to the date of documented progressive disease. The TTP_2_/TTP_1_ ratio was calculated to obtain the growth modulation index (GMI) (Von Hoff, 1998; Bonetti *et al*, 2001). Probability of progression-free and overall survival was calculated with actuarial method from the date of accrual to the date of progression, death, or last follow-up, respectively (Kaplan and Meier, 1958).

## RESULTS

### Patient characteristics

From April 1999 to December 2000, a total of 50 eligible patients were enrolled into this study from six institutions. The main characteristics of this case series are listed in Table 1

**Table 1:**
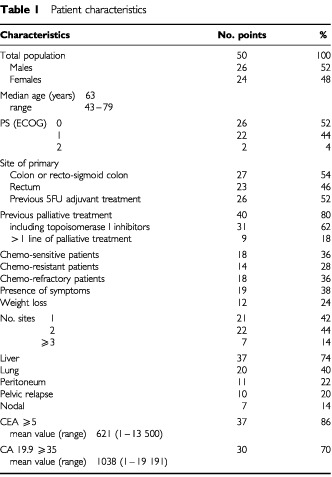
Patient characteristics

. The majority of patients had a good performance status. However, it should be noted that most of them (80%) had been exposed to chemotherapy for palliative intent (including irinotecan in 62% of them), and 18% had received more than one line of chemotherapy. On the basis of the previous exposure to chemotherapy, 18 patients (36%) were defined as chemo-sensitive, because they had showed a relapse of disease after 6 months from the end of adjuvant chemotherapy, and/or had achieved a major response with previous palliative chemotherapy, and/or the TTP_1_ was longer than 6 months; 14 patients (28%) were considered chemo-resistant, because they had a recurrence within 6 months from the discontinuation of adjuvant chemotherapy, and/or the TTP_1_ was shorter than 6 months; while 18 patients (36%) were defined as chemo-refractory, because of recurrence or progression of disease during adjuvant or palliative chemotherapy. Sites of disease were typical for these patients, with 74% of them having liver involvement. Twenty-one (42%) patients had only one site of disease, while 29 (58%) had two or more sites.

### Activity

A total of 288 cycles were delivered, with a median number of six (range, 1–12) cycles per patient. Forty (80%) patients received at least four courses, while eight cycles were delivered to 21 (42%) patients. At the time of this report, we had obtained three complete responses (CRs) and nine partial responses (PRs), for an overall response rate (RR) of 24% (95% confidence interval, 13–38%), according to intent-to-treat analysis. Three further patients showed a shrinkage of tumour burden that did not qualify for a major response, while 15 patients showed stable disease; tumour progression was clearly demonstrated in 15 patients, while five patients were not assessed (Table 2

**Table 2:**
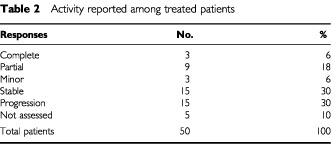
Activity reported among treated patients

). The complete disappearance of disease was achieved in three patients, who had a single lesion in the liver in two patients and lung in one patient. Two of them eventually recurred after 5.5 and 6.4 months, while the third one is still in CR at 12.4 months of follow-up. All but four responses were reached within 3 months from initial treatment, and lasted a median of 8 (range, 4.1–16.4) months. It is worth noting that three CRs and three PRs were obtained in patients pretreated with irinotecan (for a 19% RR in this subset of patients), while three PRs (RR=17%) were achieved among outright refractory patients. Extent of disease affected the probability of response, because a major tumour shrinkage was obtained in seven of 21 (33%) patients with one site of disease, compared to five out of 29 (17%) patients with two or more sites.

As of October 2001, with a median follow-up of 18 (range, 10–30) months, 40 (80%) patients had showed a further tumour progression. The GMI for these patients ranged between 0.2 and 2.5. Sixteen (40%) patients had a GMI ⩾1.33, proving that the regimen produced a significant delay of tumour growth. Interestingly, such findings appear unrelated to previous chemo-sensitivity. Indeed, it was observed in eight of 14 (57%) refractory patients, as compared to eight of 24 (33%) remaining patients. Overall median progression-free survival was 7.6 months. Eleven patients received additional treatment (capecitabine, eight patients; irinotecan, two patients; surgical resection of residual liver deposit, one patient). Thirty-three (66%) patients have died, all but one because of progressive disease. A 79-year-old patient died after two cycles because of heart failure, which might have been related to the administered chemotherapy. Overall median survival time was 13.6 months, and actuarial 1- and 2-year probabilities of survival were 55% and 9%, respectively.

### Safety

Two patients were not evaluable for toxicity, because of early discontinuation of treatment for refusal and lack of information after the first cycle. Apart from the previously mentioned early death, no toxic deaths were reported. Neutropenia was the main haematological adverse event: it was recorded during treatment in 81% of patients, and reached WHO grade 4 in 31%. Thrombocytopenia of any grade was noted in 40% of patients, but it never required platelet transfusions. Anaemia was rarely reported, and only two patients required a packed red cell transfusion (Table 3

**Table 3:**
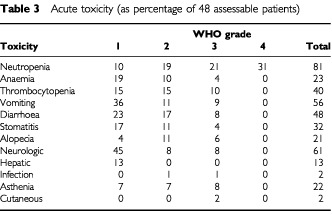
Acute toxicity (as percentage of 48 assessable patients)

). Neutropenic fever or infection occurred in two patients. As for non-haematological toxicity, gastrointestinal disturbance and peripheral neuropathy were frequently encountered. Vomiting and diarrhoea affected 56% and 48% of patients, but they reached grade 3 (no grade 4 was recorded) in 9% and 8%, respectively. Stomatitis affected about one third of patients, but it was grade 3 in 4% of them. Oxaliplatin-induced neurotoxicity was complained of by many patients, but it usually recovered before recycling without dose reduction. Ten (21%) patients complained of some fatigue attributable to treatment, which was severe in four cases. A mild and transient liver enzymes derangement was noted in 13% of patients. Due to occasional neutropenia, a reduction of all cytotoxic drugs was required in many patients; as a consequence, the mean actually delivered L-OHP dose intensity over eight cycles was 41 mg m^−2^ per week, which represented a 63% relative dose intensity (RDI). The corresponding value for TOM was 1.0 mg m^−2^ per week (67% RDI), and for FU was 336 mg m^−2^ per week (64% RDI).

## DISCUSSION

Despite the notable progress achieved in the management of advanced colorectal carcinoma, the prognosis of patients remains extremely poor. Also after the introduction into clinical practice of combination regimens, in which leucovorin-modulated FU has been associated with other active drugs such as CPT-11 or L-OHP, only a few-months delay of disease progression is gained in comparison with standard modulated-FU regimens. Furthermore, in the near future the standard adjuvant treatment of surgically resected node-positive colorectal cancer patients will likely entail a CPT-11-including regimen. Therefore, there is an urgent need for evaluating novel non-cross-resistant regimens for patients already exposed to CPT-11 plus modulated FU.

The regimen we have assessed in this study yielded a major response in 24% of patients, while 36% obtained at least a stabilisation of disease, so that tumour control was obtained in 60% of the whole series. It is also noteworthy that three CRs were achieved in this study. These CRs, although obtained in single metastatic deposits, were observed after previous exposure to topoisomerase-I inhibitors, suggesting a lack of cross-resistance between this class of drugs and oxaliplatin-based regimen. On the contrary, a weaker activity (13%) was seen among outright refractory patients. However, despite the low proportion of major responses reported among these patients, a significant improvement in tumour progression, as reflected by the GMI, was obtained in 57% of them. In addition, the progression-free and overall survival time of this heavily pretreated case series compared favourably with those reported with single-agent irinotecan after FU failure (Cunningham *et al*, 1998; Rougier *et al*, 1998). Although a formal assessment of quality of life was not performed in our study, it is likely that the delay of tumour growth obtained in many patients was paralleled by a delay of worsening of symptoms. Acute toxicity of this regimen was substantial but manageable. Indeed, despite the high frequency of severe neutropenia, neutropenic fever or infection rarely occurred, while severe non-haematological adverse events were seldom observed. However, due to occasional neutropenia, we were frequently forced to reduce the planned dosages during treatment, so impairing the ideal dose intensity of this treatment. Therefore, our regimen could probably be easily administered with a 3-weekly schedule; otherwise, using a biweekly schedule in order to keep a dose-dense treatment, a substantial reduction of all cytotoxic drugs seems advisable. Anyway, the short-lasting infusion of all cytotoxic drugs over a few hours in two separate days made this regimen convenient also for an out-patient management, because the placement of an indwelling central venous catheter and/or the use of infusional devices were not required.

Recently, a doublet regimen of L-OHP plus TOM given on the same day at full doses (130 and 3.0 mg m^−2^, respectively) every 3 weeks has been tested in a small series of advanced colorectal cancer patients (Scheithauer *et al*, 2001b). All patients had previously received one palliative FU-based regimen, which included irinotecan in 11 of them. Twelve out of 36 (33%) patients achieved a major response. However, only one of 10 truly refractory patients (progressing on first-line therapy) responded to this salvage regimen. Median progression-free and overall survival were 6.5 and >11 months, respectively. Grades 3 or 4 neutropenia affected 23% of patients. Mean dose intensity was 40.34 mg m^−2^ per week for L-OHP, and 0.986 mg m^−2^ per week for TOM.

On the other hand, the addition of L-OHP in patients progressing during a FU-based regimen has been recently addressed, with particular emphasis on the GMI measured in each patient. Indeed, while only three of 34 (9%) patients obtained a major response after the addition of L-OHP to FU, 16 (43%) of them showed a GMI ⩾1.33. The median TTP_1_ was 13 weeks, and the median TTP_2_ was 31 weeks. The median overall survival time from the start of second-line chemotherapy was 12.8 months. This observation corroborated the hypothesis that a decrease of tumour growth, rather than a significant shrinkage of tumour mass, may positively affect the survival of patients.

The results obtained in our study in terms of activity and/or control of tumour progression are in agreement with those reports. As for toxicity, the shorter recycling we have adopted may explain the greater incidence of neutropenia. However, the actually delivered dose intensity in our series was superimposable to that reported by the above mentioned study of Scheithauer *et al* (2001b) for L-OHP and TOM, despite the addition in our regimen of modulated FU. Of course, one could wonder whether this addition was needed, because of the previous exposure to FU in all patients, and because of the questionable role of FU given together with TOM.

As for the first issue, we would remember that recent *in vitro* studies have demonstrated an increased TS expression shortly after the L-OHP exposure, while the sequential administration of L-OHP before FU induced a significant decrease of TS expression, both in sensitive and resistant cell lines (Plasencia *et al*, 2000, 2001). Since a common cause of FU resistance is the over-expression of TS, it sounds wise to combine L-OHP and FU also in patients previously exposed to FU.

As for TOM and FU combination, taking into account the different binding sites, and the potentially additive or synergic inhibitory effects on TS of these two drugs, namely when given 24 h apart, we devised to assess a new regimen, in which L-OHP plus TOM administration preceded by 24 h the LFA plus FU exposure.

Finally, we should remember that, as a consequence of the increasing utilisation of CPT-11 combined with modulated-FU in the adjuvant setting as well as in front-line treatment for the advanced disease, in the next few years the medical oncologists will have to face the management of irinotecan- and FU-pretreated patients. From the present study, we conclude that our regimen represents a suitable option for some of these patients, provided that acute toxicity might be reduced by employing decreased doses or a 3-weekly schedule. However, outright refractory patients probably need a different approach with newer drugs.
